# Supplementary material for: Aggressive surgical approach with major vascular resection for retroperitoneal sarcomas
Source: PLoS One. 2025 Mar 20;20(3):e0320066. doi: 10.1371/journal.pone.0320066 (PMC11957768; doi:10.1371/journal.pone.0320066)
Supplement: S1 Table — (DOCX) [file pone.0320066.s001.docx]

**S1 Table.** **Characteristics of vascular resection**

| Characteristic | No. of patients (%) |
| --- | --- |
| Total episodes | 110 (100) |
| Surgical techniques |  |
| Primary repair | 58 (53) |
| Ligation | 19 (17) |
| Primary anastomoses | 6 (5) |
| Patch repair | 3 (3) |
| Graft reconstruction | 24 (22) |
| Location of vascular resection |  |
| IVC | 45 (41) |
| IV | 28 (25) |
| AA | 18 (16) |
| IA | 22 (20) |
| RA | 21 (19) |
| RV | 23 (21) |
| Others | 38 (35) |

Abbreviations: IVC, inferior vena cava; IV, iliac veins; AA, abdominal aortae; IA, iliac arteries; RA, renal arteries; RV, renal veins
