# Supplementary material for: Aggressive surgical approach with major vascular resection for retroperitoneal sarcomas
Source: PLoS One. 2025 Mar 20;20(3):e0320066. doi: 10.1371/journal.pone.0320066 (PMC11957768; doi:10.1371/journal.pone.0320066)
Supplement: S2 Table — (DOCX) [file pone.0320066.s002.docx]

**S2 Table. Clinicopathological and operative characteristics of patients who underwent vascular resection for primary RPSs**

| Characteristic | No vascular resection (n=92) | Vascular resection (n=71) | *p* Value |
| --- | --- | --- | --- |
| Sex (male) | 46 | 31 | 0.422 |
| Age, median (IQR) | 52 (43–61) | 50 (38–61) | 0.535 |
| Tumor size |  |  |  |
| <20 cm | 51 | 32 | 0.189 |
| ≥20 cm | 41 | 39 |  |
| Histology |  |  |  |
| Well-differentiated LPS | 22 | 9 | 0.070 |
| Dedifferentiated LPS | 16 | 16 | 0.412 |
| Leiomyosarcoma | 18 | 16 | 0.644 |
| Others | 36 | 30 | 0.687 |
| FNCLCC grade |  |  |  |
| Low | 12 | 3 | 0.053 |
| Moderate | 44 | 22 | 0.030 |
| High | 36 | 46 | 0.001 |
| Margin status (R0/1) |  |  |  |
| R0/1 | 78 | 66 | 0.107 |
| R2 | 14 | 5 |  |
| Organ resection | 27 | 36 | <0.001 |
| No. of organs resected | 1.14±0.56 | 1.31±0.53 | <0.001 |
| Transfusion | 12 | 42 | <0.001 |
| Transfusion volume (ml), median (IQR) | 400 (300–600) | 1000 (600–1500) | <0.001 |
| 30-d mortality | 2 | 3 | 0.451 |
| Complication | 4 | 5 | 0.455 |
| Length of stay (mean, d) | 15.1±11.4 | 20.7±13.9 | <0.001 |
| Length of ICU stay (mean, d) | 1.3±0.8 | 2.2±1.4 | <0.001 |
| VTE | 3 | 5 | 0.268 |

Abbreviations: IQR, interquartile range; LPS, liposarcoma; FNLCCC, Federation Nationale des Centres de Lutte Contre le Cancer; ICU, intensive care unit; VTE, venous thromboembolism
