# Supplementary figures and images for: Aggressive surgical approach with major vascular resection for retroperitoneal sarcomas
Source: PLoS One. 2025 Mar 20;20(3):e0320066. doi: 10.1371/journal.pone.0320066 (PMC11957768; doi:10.1371/journal.pone.0320066)

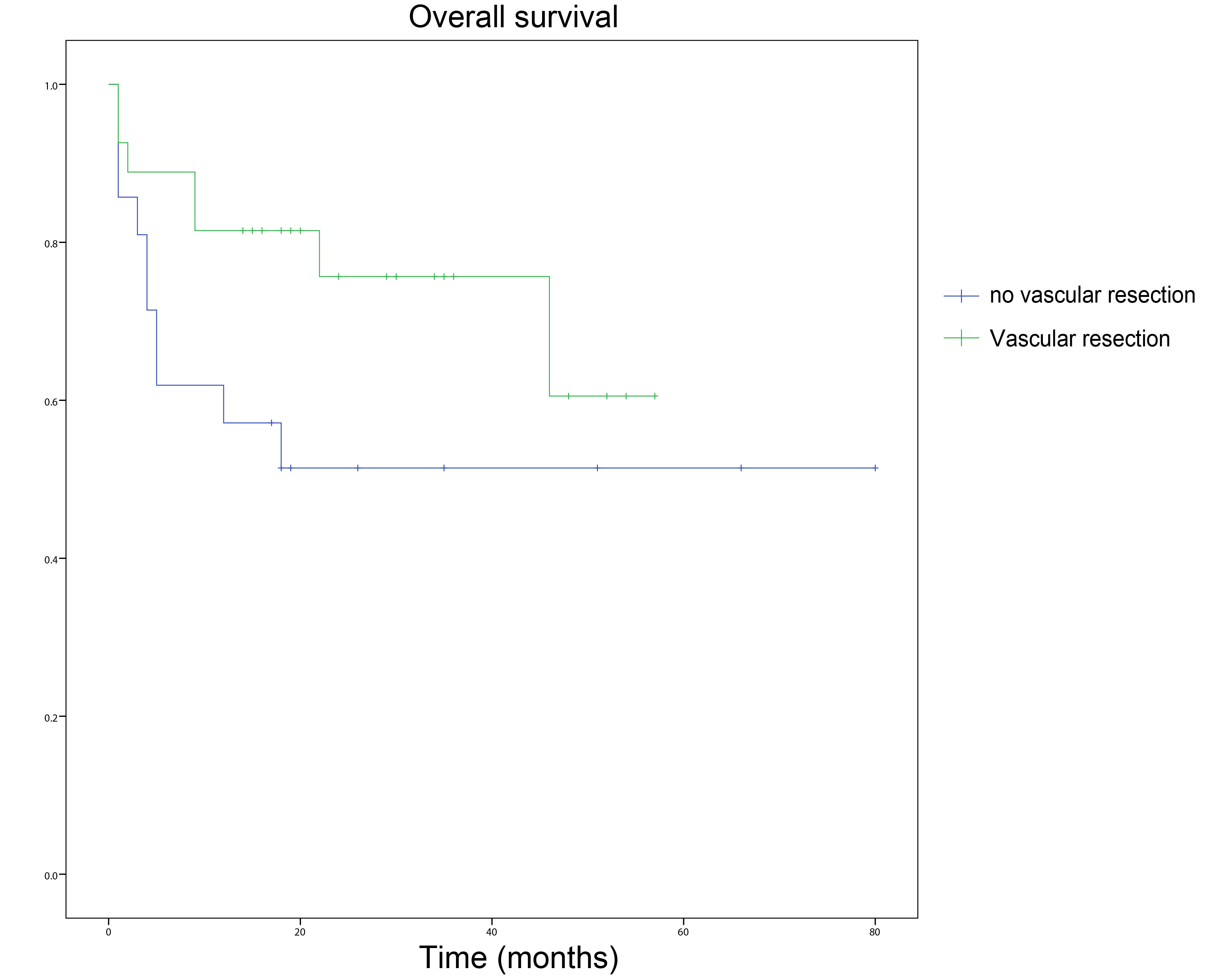

Supplement: S1 Fig — (TIF) [file pone.0320066.s003.tif]

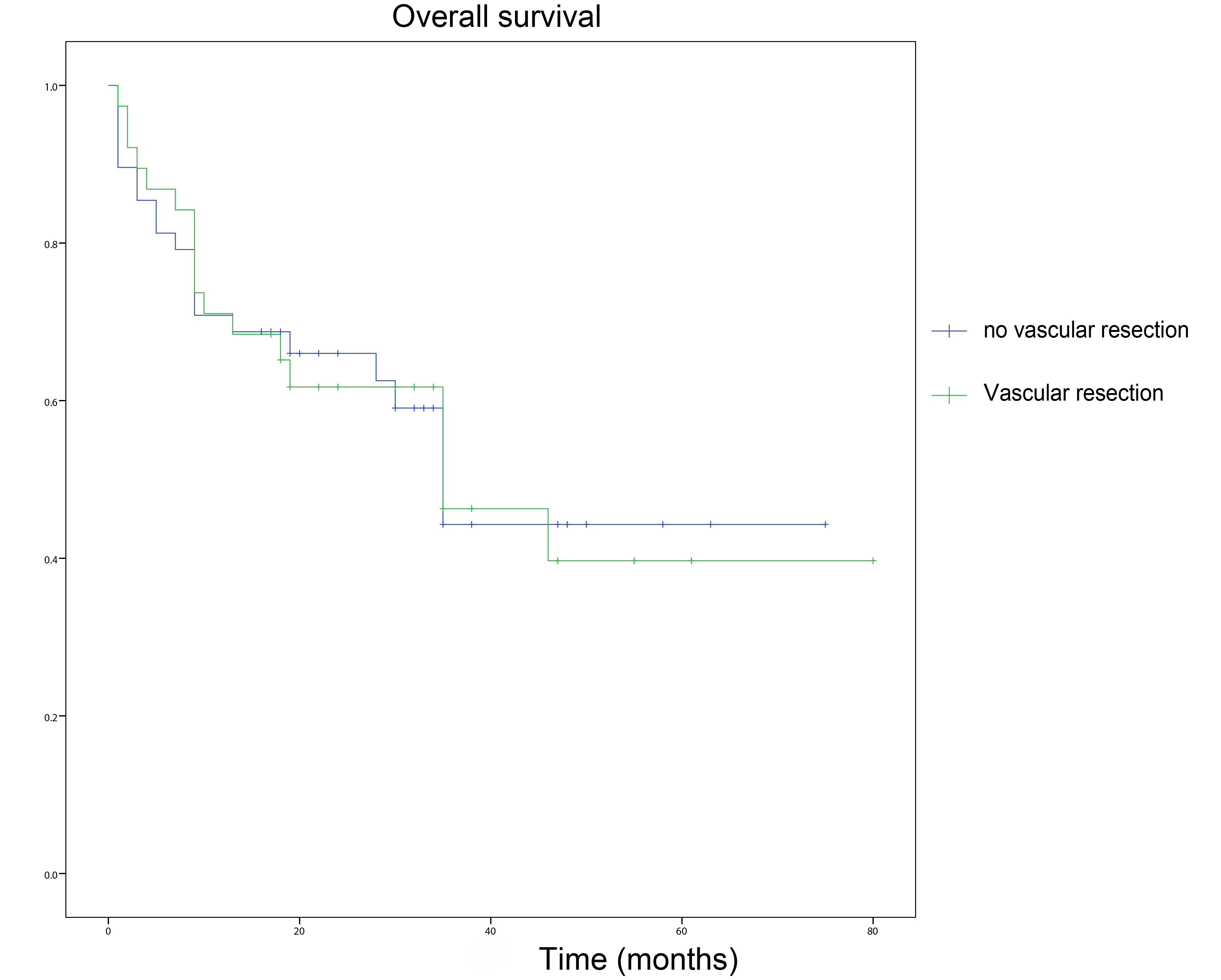

Supplement: S2 Fig — (TIF) [file pone.0320066.s004.tif]

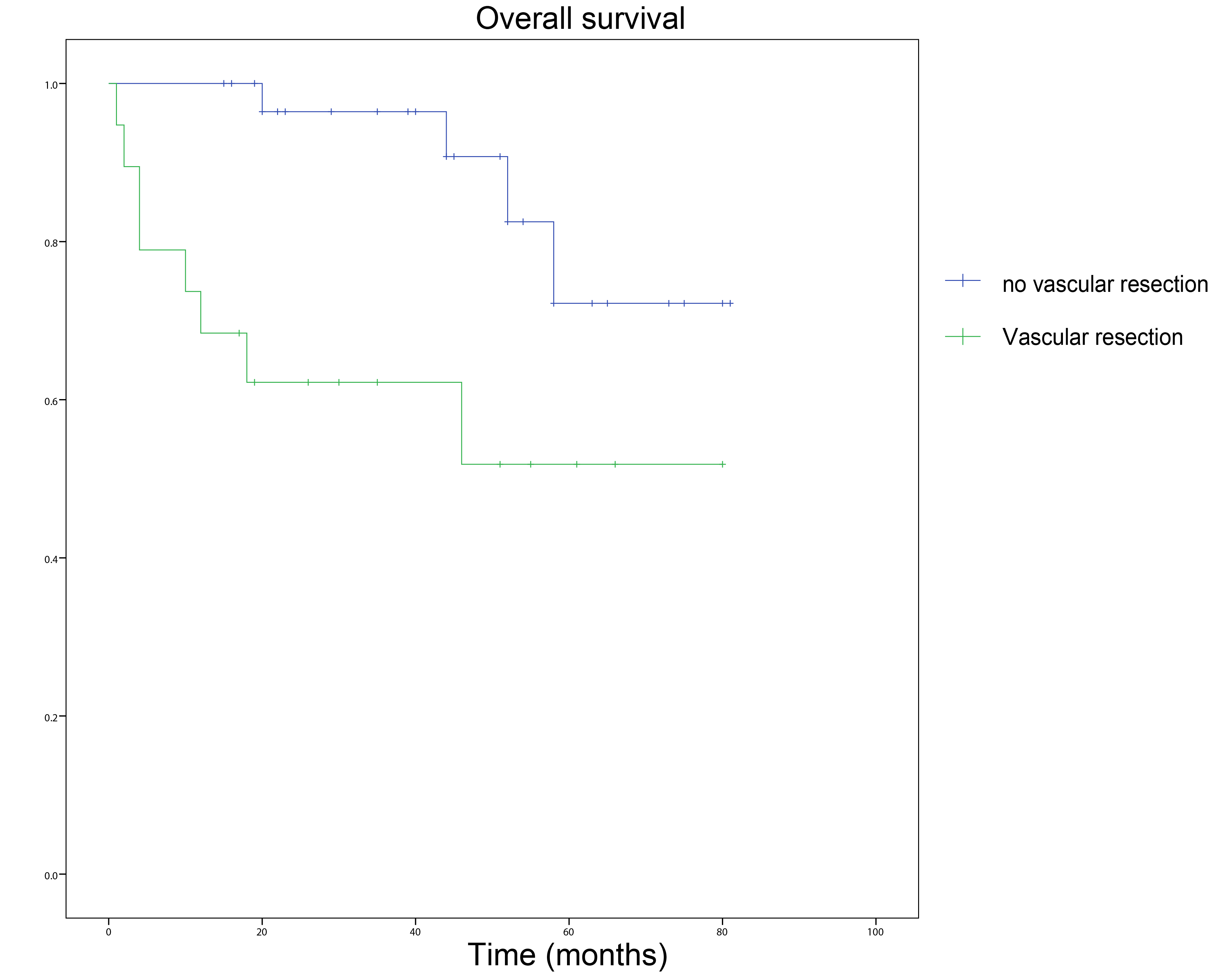

Supplement: S3 Fig — (TIF) [file pone.0320066.s005.tif]

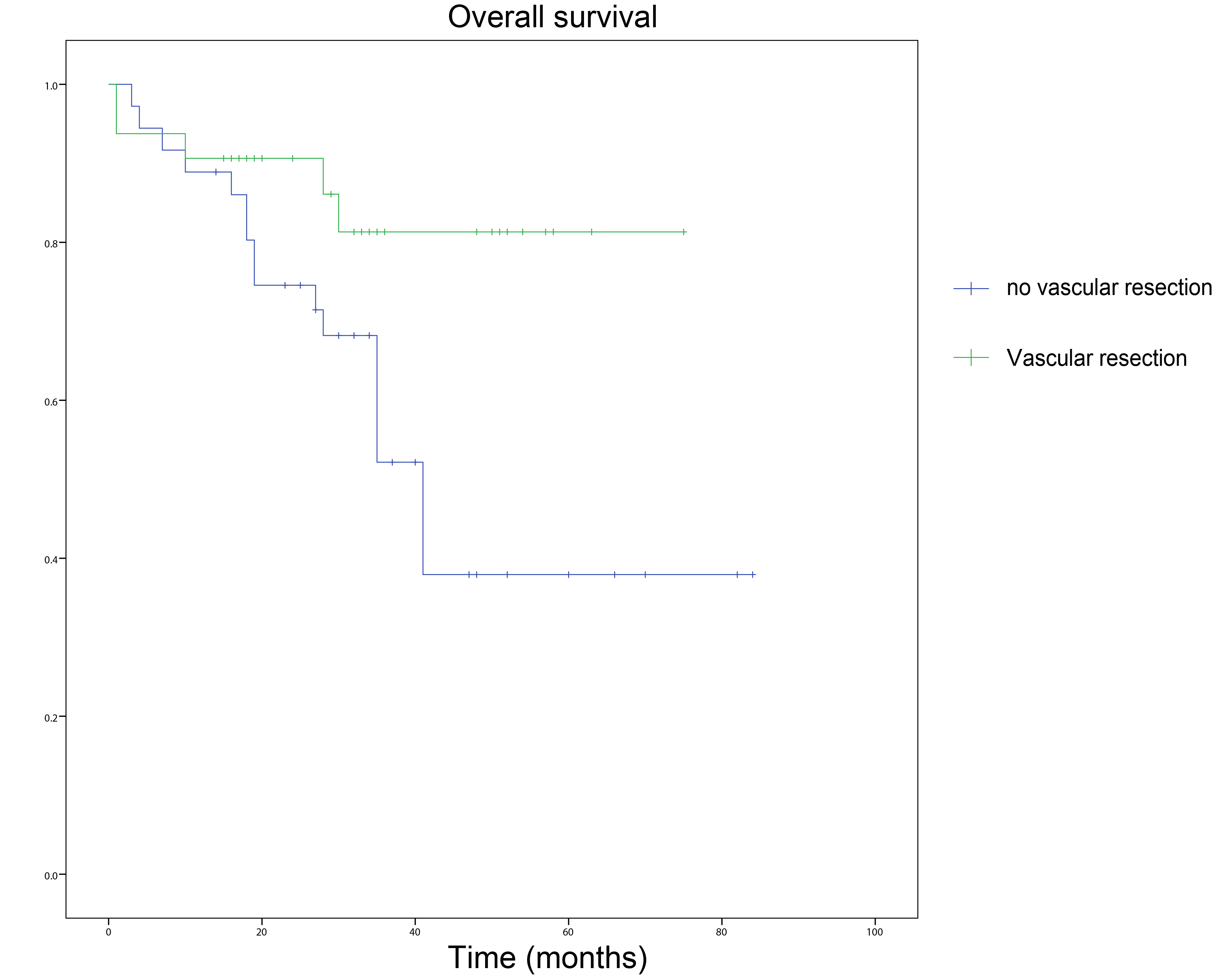

Supplement: S4 Fig — (TIF) [file pone.0320066.s006.tif]
