# Supplementary material for: Aggressive surgical approach with major vascular resection for retroperitoneal sarcomas
Source: PLoS One. 2025 Mar 20;20(3):e0320066. doi: 10.1371/journal.pone.0320066 (PMC11957768; doi:10.1371/journal.pone.0320066)
Supplement: Attachment — Submitted filename: Comments from the reviewers.docx [file pone.0320066.s008.docx]

**Reviewer’s Comments**

Article: Aggressive surgical approach with major vascular resection for retroperitoneal sarcomas.

This study evaluates the safety and outcomes of aggressive en bloc resection with vascular involvement in retroperitoneal sarcoma (RPS). It demonstrates that vascular resections improve long-term survival without increasing morbidity or mortality. The findings support curative-intent vascular resections as a feasible strategy for patients with RPS and vascular involvement and are highly relevant for improving survival outcomes and patient care strategies. The manuscript addresses an important and underexplored topic- vascular resections in retroperitoneal sarcoma (RPS), which has significant implications for surgical oncology practice.

This study has used a prospectively maintained database to ensure a robust dataset for analysis. It uses subgroup analyses, including reconstruction techniques, to enhance the depth of the study and allow nuanced conclusions. The manuscript is well-organized, with a logical flow from background to discussion. The data presented in tables and Kaplan-Meier curves is clear. It provides valuable evidence supporting the safety and feasibility of en bloc vascular resections and adds to existing literature by highlighting the potential of aggressive surgical approaches in improving long-term outcomes.

However, I have come across certain issues with the manuscript that need to be addressed to be accepted for publication in this journal.

Some of the major changes are:

1. Although the rationale for the study has been discussed in the introduction section, this section should present a more detailed comparison with recent studies on vascular resections in RPS. Also, the authors should consider highlighting the advancements or gaps addressed by this study.
2. The authors should consider providing more context on how the analysis of baseline differences between vascular and non-vascular resection groups occurs (like in the case of propensity score matching).
3. The authors should elaborately discuss why patients with higher FNCLCC grades were more prevalent in the vascular resection group.
4. Please discuss the implications of higher blood loss, VTE rates, and extended ICU stays for patient outcomes in detail.
5. The authors should highlight the potential strategies to mitigate these risks in clinical practice address the lack of significant differences in overall survival between primary repair and reconstruction techniques and propose hypotheses for these findings.
6. The authors should consider discussing the lack of pathology-confirmed vascular invasion and its impact on findings in a more elaborate manner while discussing the limitations.
7. The authors should mention if any efforts were made to control institutional biases due to the single-center nature of the study.
8. In the figures and tables section, the authors should add a summary table comparing key outcomes across reconstruction techniques for easier reference. Please ensure that all figures (e.g., Kaplan-Meier plots) are annotated with clear legends and relevant *p*-values for subgroup comparisons.
9. In the discussion section, the authors should include recommendations for multicenter studies or randomized trials to validate findings. They should suggest areas for innovation in surgical techniques or perioperative management to further improve outcomes.

I also found a few minor issues in the manuscript and working on them can improve the manuscript:

1. The authors should reassess sentence structures in the abstract and conclusion to make the findings more concise and impactful.
2. Inconsistent use of terminologies in the manuscript reduces the clarity of the manuscript and might confuse the readers. Therefore, the authors should ensure the use of terminologies consistently throughout the manuscript (for example the authors should either use “en bloc resection” or “aggressive surgical approach” and these terms should not be used interchangeably).

Overall, the manuscript seems promising and has substantial merit in contributing to the field. I believe that addressing these points mentioned above will further strengthen the study’s impact and clarity and make it suitable for acceptance to the journal.
